# PLA-Based Electrospun Nanofibrous Mats Towards Application as Antibiotic Carriers: Processing Parameters, Fabrication and Characterization

**DOI:** 10.3390/pharmaceutics17050589

**Published:** 2025-04-30

**Authors:** Evi Christodoulou, Anastasia Chondromatidou, Nikolaos D. Bikiaris, Evangelia Balla, Marilena Vlachou, Panagiotis Barmpalexis, Dimitrios N. Bikiaris

**Affiliations:** 1 Laboratory of Polymer Chemistry and Technology, Department of Chemistry, Aristotle University of Thessaloniki, 54124 Thessaloniki, Greece; evicius@gmail.com (E.C.); anastasiahon24@gmail.com (A.C.); euagelia226@gmail.com (E.B.); 2 Laboratory of Pharmaceutical Technology, Division of Pharmaceutical Technology, School of Pharmacy, Faculty of Health Sciences, Aristotle University of Thessaloniki, 54124 Thessaloniki, Greece; nbikiaris@gmail.com (N.D.B.); pbarmp@pharm.auth.gr (P.B.); 3 Section of Pharmaceutical Technology, Department of Pharmacy, School of Health Sciences, National and Kapodistrian University of Athens, Panepistimioupoli-Zografou, 15784 Athens, Greece; vlachou@pharm.uoa.gr

**Keywords:** polymeric nanofibers, electrospinning, poly(lactic acid) (PLA), pentaerythritol, transdermal drug delivery, levofloxacin (LEV), antibiotic carriers

## Abstract

**Background/Objectives**: Polymeric nanofibers are promising platforms for skin treatment applications due to their large surface area and high porosity, which promote enhanced drug delivery. This study aimed to develop and compare poly(lactic acid)-based (PLA) nanofibrous mats, using linear PLA and a star-like PLA-pentaerythritol (PLA-PE) copolymer, as carriers for transdermal delivery of the antibacterial agent levofloxacin (LEV). **Methods**: Electrospinning was employed to fabricate nanofibers from PLA and PLA-PE solutions. Spinning parameters and polymer concentrations (10% *w*/*v* PLA and 20% *w*/*v* PLA-PE) were optimized to produce uniform fibers. LEV was loaded at 10% and 20% *w*/*w*. A sum of complementary characterization techniques, including scanning electron microscopy (SEM), infrared spectroscopy (FTIR), X-ray diffraction (XRD), and differential scanning calorimetry (DSC), were applied to comparatively investigate the fibers’ morphology, structural properties, and crystallinity. Drug loading, porosity, degradation, and in vitro release profiles were evaluated. **Results**: PLA-PE nanofibers demonstrated smaller diameters and higher porosity (up to 90.1%) compared to PLA (82.4%), leading to enhanced drug loading (up to 34.78%) and faster degradation (55% vs. 43% mass loss over 60 days). Drug release exhibited a biphasic profile with an initial burst followed by sustained release. PLA-PE formulations released up to 60.2% LEV, compared to 38.1% for PLA counterparts. **Conclusions**: The star-like PLA-PE copolymer enhances nanofiber properties relevant to the desired application, including porosity, degradation rate, and drug release. These findings suggest that PLA-PE is a promising material for developing advanced transdermal antibiotic delivery systems.

## 1. Introduction

Transdermal drug delivery systems (TDDS) have emerged as a promising alternative to conventional drug administration methods, offering numerous advantages over other conventional routes, such as improved patient compliance, reduced systemic side effects, and enhanced therapeutic efficacy [1,2,3]. By passing the gastrointestinal tract, TDDS can prevent first-pass metabolism and provide sustained drug release, leading to more stable plasma drug concentrations. Advances in nanotechnology have allowed for the development of various types of TDDS towards more effective and safer treatments [4,5,6]. Among them, polymeric nanofibers have gained prominence due to their unique properties, such as the large surface area available, high porosity, improved bioavailability, and enhanced treatment adherence by delivering drugs at a controlled rate over time to the site of action [7,8,9,10].

A broad range of methods have already been explored and are currently accessible for fabricating nanofibrous structures, with electrospinning (ES) standing out and attracting considerable attention due to its simplicity, cost-effectiveness, and ability to produce fibers in the submicron size range using a diverse selection of fiber-forming polymeric materials. Electrospun nanofibers have been extensively explored across a spectrum of diverse applications [11], from energy storage [12] to sensor technology [13], and of course drug delivery [14,15]. This versatile and efficient technique involves the application of a high-voltage electric field (usually between 1 to 30 kV) to a polymer solution or melt, resulting in the formation of ultrafine fibers that are collected in a nonwoven mat [16]. It also facilitates the incorporation of active agents, rendering it a highly promising approach for biomedical applications, such as wound dressings and tissue engineering [17]. The process parameters, including polymer concentration, solvent type, voltage, and flow rate, play a crucial role in determining the final fiber morphology and porosity, characteristics that can significantly influence drug release kinetics and cellular adhesion/proliferation [18].

Among the variety of polymers employed for the preparation of nanofiber-based drug carriers, poly(lactic acid) (PLA) has emerged as an attractive option due to its biodegradability, biocompatibility, and excellent mechanical strength [19,20,21,22]. As a thermoplastic aliphatic polyester derived from renewable sources, PLA has been extensively utilized in biomedical applications [23]. Its degradation products, primarily lactic acid, are naturally metabolized by the human body, thereby minimizing toxicity concerns. Electrospinning PLA-based nanofibers results in highly porous structures with large surface area-to-volume ratios, enabling controlled and sustained drug release [24]. The functionality of PLA-based electrospun fibers can be further enhanced through the incorporation of copolymers [25], plasticizers [26,27], and nanoparticles [28,29,30], which allow for improved mechanical properties, finely tuned biodegradation rates, and tailored release kinetics. Recently, star-shaped PLA materials, characterized by a branched architecture, have attracted growing interest due to their superior rheological, mechanical, and biomedical properties, which exceed those of conventional linear polymers [31]. Unlike their linear counterparts, star-shaped PLA copolymers possess a higher number of terminal groups, resulting in enhanced solubility and a significant influence on hydrodynamic volume.

In the context of this study, levofloxacin (LEV), a broad-spectrum fluoroquinolone antibiotic, was selected as the model drug for incorporation into electrospun PLA fibers. LEV is highly effective against both Gram-positive and Gram-negative bacteria, including *Streptococcus pneumoniae* and meticillin-sensitive *Staphylococcus aureus* (MSSA). Its efficacy against antibiotic-resistant bacterial strains renders it particularly valuable for developing advanced wound dressings for severe bacterial infections. Research findings have demonstrated that incorporating LEV into electrospun polymeric nanofibers can enhance its therapeutic efficiency by enabling controlled and localized drug delivery, while minimizing systemic side effects [32,33,34]. By modifying the solution composition and electrospinning parameters, the physicochemical and morphological properties of PLA-based fibers can be tailored to optimize their performance in biomedical applications [35,36].

Our study aims to fabricate and comparatively characterize electrospun nanofibrous mats based on both linear poly(lactic acid) (PLA) and a star-like PLA-pentaerythritol (PLA-PE) copolymer [37,38]. While PLA is a well-established biomaterial, the introduction of the star-shaped PLA-pentaerythritol (PLA-PE) copolymer is hypothesized to offer control over fiber morphology, degradation rate, and, subsequently, drug release kinetics. Therefore, a key focus of this work is to systematically compare the physicochemical properties of PLA and PLA-PE nanofibers, investigating the impact of the pentaerythritol modification on the final fiber performance. Furthermore, we will assess key parameters such as porosity, degradation rates, and in vitro drug release profiles of both PLA and PLA-PE matrices. By optimizing the electrospinning process and incorporating the antibacterial drug LEV, we aspire to develop an effective transdermal drug delivery system that overcomes current limitations.

## 2. Materials and Methods

### 2.1. Materials

Levofloxacin (LEV, C_18_H_20_FN_3_O_4_) was kindly donated by Pharmathen Pharmaceutical Company (Athens, Greece). The polyesters—neat PLA and its star-like copolyester with pentaerythritol (PLA-PE)—were previously synthesized and thoroughly studied by our group, as described in detail in the original publication [37]. The PLA had an Mn of 55.5 g/mol and a PDI of 2.18, while the PLA-PE copolymer had an Mn of 25.0 g/mol and a PDI of 1.81. Table 1 below summarizes the important values with respect to their molecular weight and thermal behavior. Chloroform (CHCl_3_) (≥99.8%, ACS reagent grade), used as solvent, was purchased from Aldrich Chemical Co. (Stainheim, Germany). All other reagents utilized were of analytical or pharmaceutical grade.

### 2.2. Solution Preparation and Electrospinning Process

A series of PLA and PLA-PE of varying concentrations (5, 10, 15 and 20% *w*/*v*) were prepared and tested by dissolving the appropriate amount of each material in chloroform (CHCl_3_). The prepared solutions were loaded into 6 mL syringes and electrospun using a horizontal system with a cylindrical collector covered by aluminum foil (Fluidnatek LE10) at RT. A range of high voltage (20–22 kV) was applied to the tip of the needle to force the solutions through the needle, forming randomly oriented fibers. Two flow rates (750 and 1000 μL/h), two different rotating speeds of the collector (500 and 1000 rpm), and two different needle-to-collector distances (10 and 12 cm) were tested. The prepared electrospun nanofibers were kept at RT and in ambient conditions until further characterization.

### 2.3. Structural-Morphological Characterization & In Vitro Drug Release Testing

#### 2.3.1. Attenuated Total Reflection Fourier Transform Infrared (ATR-FTIR) Spectroscopy

ATR-FTIR spectra were acquired using an IRTracer-100 spectrometer (Shimadzu, Kyoto, Japan) fitted with a QATR^TM^ 10 single-reflection accessory and a diamond crystal. The spectra were collected over the range 450 to 4000 cm^−1^ at a resolution of 2 cm^−1^ (a total of 16 co-added scans), while the baseline was corrected and converted into absorbance mode.

#### 2.3.2. Differential Scanning Calorimetry (DSC)

DSC measurements were carried out using a Pyris Diamond DSC instrument (Perkin-Elmer, Dresden, Germany). Calibration was performed using zinc and indium standards. Nitrogen flow (50 mL/min) was applied to provide a constant thermal blanket within the DSC cell. Approximately 5 ± 0.1 mg of each sample (neat PLA and PLA-polyol formulations) was sealed in aluminum pans and subjected to heating 50 °C above its respective melting point at a rate of 20 °C/min to eliminate prior thermal history. Two subsequent heating scans of the quenched samples were recorded to observe the melting temperatures (T_m_). In the first scan, the samples were heated from 20 to 250 °C with a heating rate of 20 °C/min, while in the second scan, they were cooled to 0 °C with a cooling rate of 20 °C/min and heated again to 250 °C with a heating rate of 20 °C/min.

#### 2.3.3. X-Ray Diffraction (XRD)

X-ray diffraction (XRD) patterns were obtained using a MiniFlex 600 instrument (Rigaku Co., Tokyo, Japan) operating with CuKα radiation (λ = 0.154 nm). The scans were conducted over a 2θ range of 5° to 45° at a rate of 1° per minute.

#### 2.3.4. Scanning Electron Microscopy (SEM)

Scanning electron microscopy (SEM) was used to investigate samples’ morphology and microstructure. To ensure adequate conductivity, the samples were initially carbon-coated, before being examined with a JEOL (JMS-840A) scanning microscope (Jeol Ltd., Akishima, Japan). The SEM analysis was performed using an accelerating voltage of 20 kV, a probe current of 45 nA, and a counting time of 60 s.

#### 2.3.5. Porosity Calculation

Porosity was determined by assessing the weight difference between the electrospun sample and a theoretical solid sample of the same dimensions, measured using a precision thickness gauge. % Porosity (P) was then determined by using Equation (1):

(1) $$\%\;P=\frac{M_{1}-M_{2}}{M_{1}}\times 100$$

where P is the porosity, M_1_ is the mass of a fully dense sample, and M_2_ is the mass of the electrospun mat. All samples were of the same dimensions for comparative reasons.

#### 2.3.6. In Vitro Degradation Testing

For the degradation test of each of the selected mats, 10 × 60 × 0.2 mm sheets of nanofibrous material were prepared. Each specimen was placed in 10 mL phosphate-buffered saline (PBS, pH 7.4). The specimens were stored in an incubator at 100 rpm, 37.0 ± 0.5 °C up to 60 days. The PBS solution was replaced weekly. All specimens were washed with distilled water to remove residual solution, and the wet weight of each specimen was measured. To measure the dry weight, specimens were dried in a vacuum oven for 48 h at 40 °C and then weighed. The difference between the initial mass (W_o_) and the mass after the immersion (W_t_) provided the initial mass of the degraded sample; thus, the (%) weight loss of the sample was derived using the following equation:

(2) $$\%\;\mathrm{Mass}\;\mathrm{Loss}=\frac{W_{o}-W_{t}}{W_{o}}\times 100$$

#### 2.3.7. Drug Loading and In Vitro Drug Release

Percentage drug loading (DL) and entrapment efficiency (EE) were estimated by dispersing 10 mg of the LEV-loaded mats in methanol. The dispersions were stirred overnight and filtered using PTFE hydrophobic filters of 0.45 μm pore size. The LEV content was determined using a Shimadzu HPLC prominence system (Kyoto, Japan), consisting of a degasser (DGU-20A5, Kyoto, Japan), a liquid chromatograph (LC-20 AD, Kyoto, Japan), an autosampler (SIL-20AC, Kyoto, Japan), a UV/Vis detector (SPD-20A, Kyoto, Japan), and a column oven (CTO-20AC, Kyoto, Japan). A CNW Technologies Athena C18, 120 A, 5 μm, 250 mm × 4.6 mm analytical column was used, and the analysis was performed at 25 °C. The mobile phase consisted of methanol/water (acidified with phosphoric acid at final pH = 3.0) 40/60 *v*/*v*, at a flow rate of 1.0 mL/min. UV detection was performed at 295 nm. The injection volume was 10 μL. The calibration curve was created by diluting a stock methanol solution of 500 ppm LEV to concentrations of 0.01, 0.025, 0.05, 0.1, 0.25, 0.5, 1.0, 2.5, 5.0, 10.0, 25.0, and 50.0 ppm. % DL and EE values were calculated using the following equations:

(3) $$\%\;\mathrm{DL}=\frac{Weight\;of\;LEV\;in\;nanofibers}{Weight\;of\;nanofibers}\times 100$$

(4) $$\%\;\mathrm{EE}=\frac{Weight\;of\;LEV\;in\;nanofibers}{Initial\;weight\;of\;LEV}\times 100$$

The in vitro release studies were conducted in a DISTEK Dissolution Apparatus II (North Brunswick, NJ, USA) equipped with an autosampler. Dissolution was performed at 37 ± 0.5 °C, and the rotation speed was set at 50 rpm. The dissolution medium was 300 mL of PBS at pH = 7.4, with 0.1% *v*/*v* of Tween 20 (used to maintain perfect sink conditions). Two milliliters of aqueous solution were withdrawn from the release media at predefined time intervals and quantified via the HPLC method previously described.

## 3. Results and Discussion

### 3.1. Preliminary Evaluation of Process Parameters and Morphology

**Figure 1:**
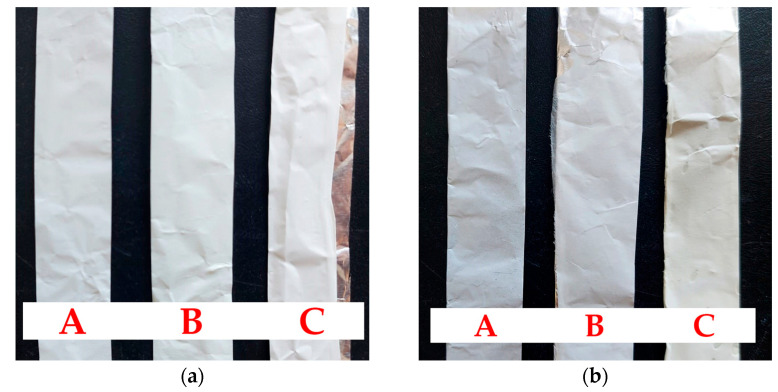
Images of the prepared (**a**) PLA and (**b**) PLA-PE electrospun mats. In both cases: (A) neat polymer, (B) 10% *w*/*w* LEV-loaded, and (C) 20% *w*/*w* LEV-loaded.

Preliminary studies focused on identifying the key parameters that could afford consistent fiber diameters, minimal defects, and generally stable electrospinning behavior. Parameters such as polymer concentration, voltage, flow rate, and working distance were systematically varied. SEM analysis was employed to assess fiber morphology and overall quality, revealing that PLA-PE matrices exhibited generally higher porosity, but also increased bead formation compared to neat PLA, which will be further discussed later. During the optimization of electrospinning parameters, PLA solutions exhibited more consistent fiber formation in general, while PLA-PE solutions tended to produce fibers with higher porosity and increased bead formation, indicating a significant influence of the pentaerythritol modification on the electrospinning behavior. Table 2 presents the various samples tested used to optimize the fabrication process:

Based on the evaluation of the above samples, the solutions with optimized electrospinning stability and fiber-forming ability were selected. Four LEV-loaded samples were then prepared with the addition of 10 and 20% *w*/*w* LEV (drug-to-polymer ratio) and stirred for 30 min at RT. Table 3 below summarizes the solution concentrations and process parameters of the final prepared nanofibrous mats, and Figure 1 presents an overview of the prepared mats.

It is crucial to note that although chloroform was employed as the solvent in our study due to its high volatility and excellent solubilizing capacity for PLA-based copolymers, it raises an environmental and safety flag, posing a limitation in terms of sustainability and potential industrial scalability. Future work needs to focus on evaluating greener alternatives, such as ethyl acetate, acetone, or their mixtures, towards nanofibers with comparable quality and improved safety profiles.

### 3.2. Morphological Analysis

#### 3.2.1. SEM

**Figure 2:**
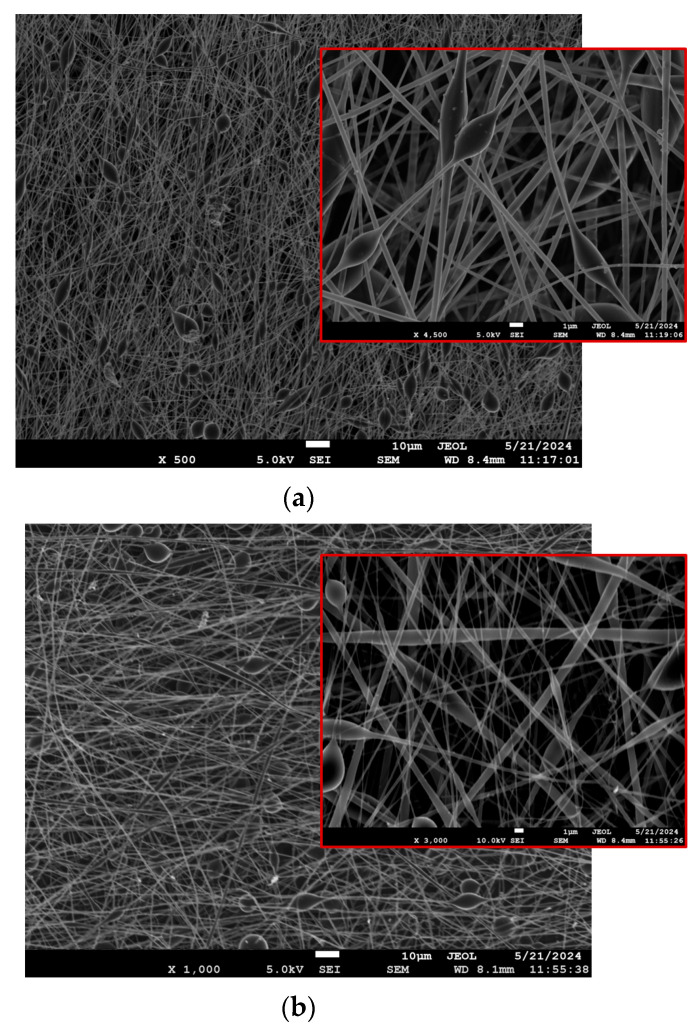

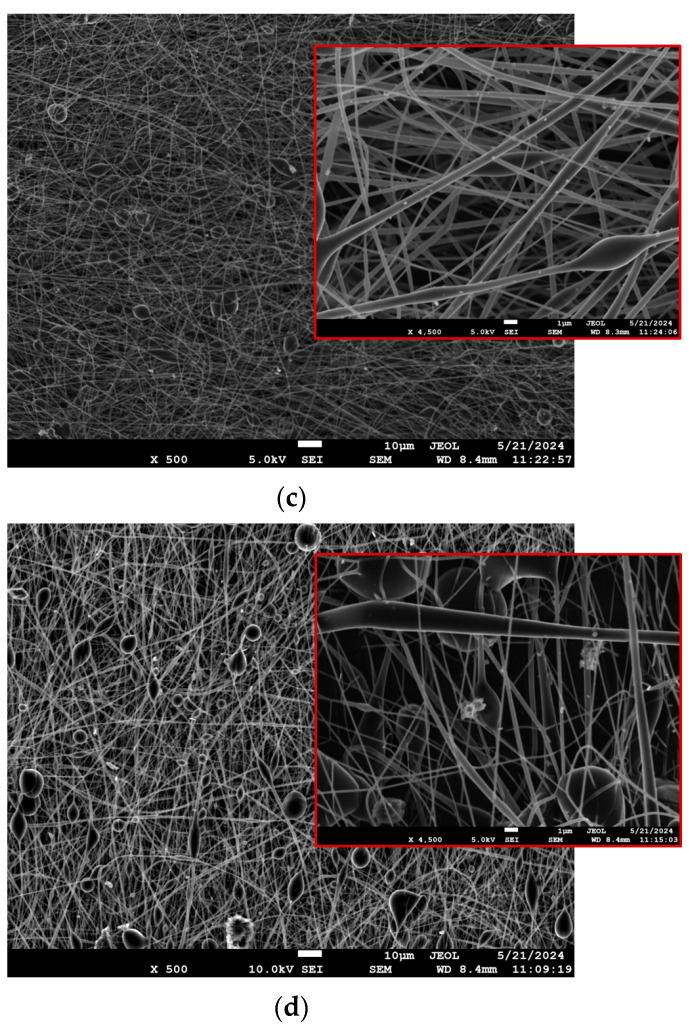
SEM micrographs of the prepared LEV-loaded nanofibrous mats at two different magnifications: (**a**) S1 (PLA10LEV10), (**b**) S2 (PLAPE20LEV10), (**c**) S3 (PLA10LEV20), and (**d**) S4 (PLAPE20LEV20).

A comparative SEM analysis of the two matrices highlighted the distinct morphological differences, with PLA fibers showing a more uniform structure, while PLA-PE fibers demonstrate increased porosity and bead formation, particularly evident at higher magnifications. In all cases, the SEM images of the LEV-loaded electrospun nanofibers revealed a randomly oriented, non-woven mat structure with average fiber diameters ranging from 334 to 665 nm (as previously presented in Table 3). As evident from Figure 2a–d, the lower magnification images show a mostly uniform fiber distribution across the sample, while the higher magnification provides a more detailed view of individual fibers, where the presence of beads or spindle-like formations can be clearly observed, especially in the case of PLA-PE matrices. The latter could possibly be attributed to the lower molecular weight of PLA-PE copolymer, which leads to a looser polymer cohesion during the fiber fabrication and increases the bead formation potential [39]. Moreover, a significant increase in bead formation can be noticed when the LEV concentration is raised to 20% *w*/*w*, indicating that the presence of levofloxacin may disrupt the polymer matrix, affecting jet stability and leading to phase separation or polymer–drug aggregation [40].

#### 3.2.2. Porosity Calculations

**Figure 3:**
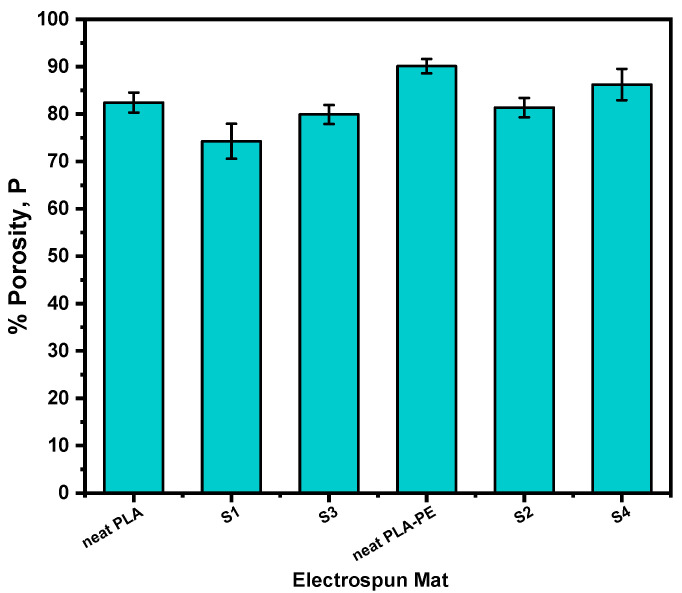
Bar chart diagram providing the porosity (P) values for all fabricated electrospun mats (neat and LEV-loaded).

The porosity of the nanofibers could significantly affect the drug release kinetics, as it is directly linked to the surface area, and thus the network permeability and the extent of drug–polymer interactions [41]. Typically, higher porosity can facilitate faster drug release by increasing the available surface for dissolution, whereas lower porosity can hinder/sustain the drug release by hindering drug mobility and/or water penetration. Figure 3 illustrates the percentage porosity of neat PLA, neat PLA-PE, and the LEV-loaded electrospun nanofibers. It can be observed that neat PLA-PE nanofiber matrix exhibits the highest porosity (90.12%), indicating that the copolymerization with pentaerythritol in PLA-PE results in a more porous structure compared to neat PLA (82.43%). Most likely, the polyol pentaerythitol introduces additional branching and crosslinking points within the polyester chain, leading to a looser macromolecular arrangement and a reduced chain-packing ability, which leads to the formation of more free spaces, resulting in a more open and interconnected porous architecture [42].

Upon loading with 10% *w*/*w* LEV, a decrease in porosity is observed compared to the neat samples (74.26 and 81.37% for S1 and S2, respectively), which can be attributed to the drug molecules occupying some of the available free spaces within the nanofibers, resulting to a more compact network with reduced pore volumes [43]. However, by further increasing the drug amount to 20% *w*/*w* LEV, a slight increase (79.91 and 86.24% for S3 and S4, respectively) is occurring.

### 3.3. Structural Investigation (ATR-FTIR)

**Figure 4:**
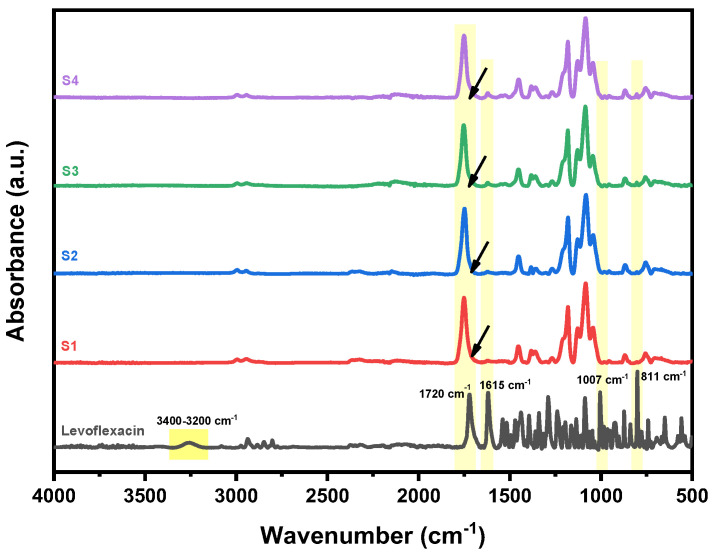
ATR-FTIR spectra of S1–S4 nanofibers samples compared to pure levofloxacin.

The structure of the produced LEV-loaded nanofibers was assessed using ATR-FTIR spectroscopy to investigate any possible interactions and structural alterations after the incorporation of the drug. In Figure 4, the spectra of S1–S4 nanofibers are presented against that of pure levofloxacin. As can be seen, pure LEV exhibits characteristic absorption peaks corresponding to its functional groups, including a broad O–H stretching peak at 3400–3200 cm^−1^ due to hydrogen bonding and N–H stretching peak from the secondary amine, and a strong C=O stretching peak at 1720 cm^−1^ from the carboxyl and ketone groups. Additional characteristic peaks include C–F stretching at 1007 cm^−1^, C=N and C=C stretching of the quinolone core at 1615 cm^−1^, and aromatic C–H bending at 811 cm^−1^. In the of the drug-loaded electrospun fibers, the spectra confirm the successful incorporation of LEV. As can be seen, the characteristic peaks of pure LEV are observed in all nanofiber samples (S1–S4), indicating the presence of the drug within the fibers. The intensity of the LEV peaks is higher in S3 and S4 (20% drug loading) compared to S1 and S2 (10% drug loading), which is consistent with the increased drug concentration. The presence of the drug’s characteristic functional groups in the nanofibers indicates that its structure is preserved during the electrospinning process. Shifts or changes in peak intensities, for example the broadening of the characteristic C=O stretching peak of the polyester backbone at 1750 cm^−1^, suggest potential drug–polymer interactions such as hydrogen bonding and molecular dispersion.

### 3.4. Crystalline Structure Assessment (XRD)

**Figure 5:**
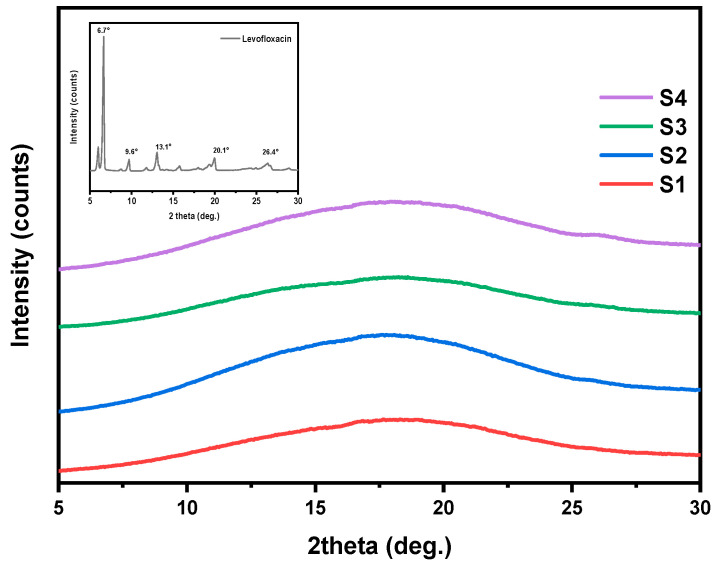
XRD patterns of pure LEV and S1–S4 nanofibrous mats.

The physical state of the drug significantly influences its dissolution and release kinetics; therefore, the crystalline morphology of the final formulation is a critical parameter to assess. In general, a drug in amorphous state typically leads to faster dissolution rates and improved bioavailability. Figure 5 summarizes the XRD diffraction patterns of the pure drug and the fabricated nanofiber mats over the region of 2*θ* = 5–30°. As can be seen, pure levofloxacin is a highly crystalline compound, with several distinct diffraction peaks at 2*θ* = 6.6°, 9.6°, 13.1°, 20.1°, and 26.4° [44,45] (Figure 5, inset plot). In the case of the LEV-loaded nanofibers (S1, S2, S3, and S4), all XRD patterns exhibit broad amorphous halos and the sharp diffraction peaks of LEV are no longer present, indicating that the drug is well-dispersed in an amorphous state within the nanofibers [46]. These results suggest that either the electrospinning fabrication process itself or the developed drug–polymer interactions disrupted the crystalline structure of LEV.

The amorphous dispersion of LEV is expected to enhance its solubility and dissolution rate. However, it is important to notice that amorphous forms can be thermodynamically unstable over time and may recrystallize under certain storage conditions, potentially affecting drug efficacy and release behavior. Further investigation into the long-term physical stability of the amorphous drug within the nanofiber matrix is thus required.

### 3.5. Investigation of Thermal Transitions (DSC)

**Figure 6:**
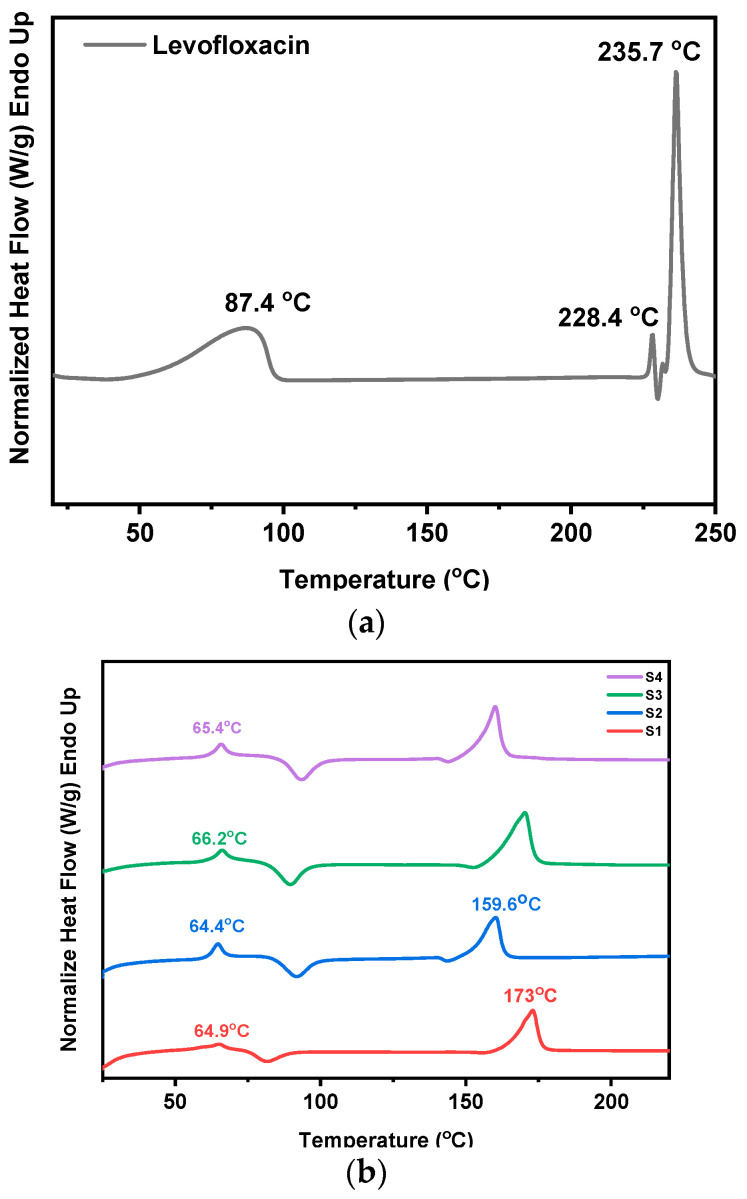
DSC thermograms of (**a**) pure LEV and (**b**) S1–S4 nanofibrous mats.

To assess the thermal behavior of the prepared LEV-loaded nanofibers and identify possible interactions between the drug and the polymers, DSC measurements were performed. Figure 6a shows the recorded DSC thermogram of pure levofloxacin, exhibiting three thermal events. The first endothermic transition occurs at approximately 87.4 °C, which most likely corresponds to the loss of water (e.g., dehydration of crystalline hydrates) or a relaxation phenomenon. The second endothermic peak observed at 228.4 °C, is attributed to a thermal event linked to a possible phase change, i.e., a polymorphic transition or pre-melting degradation. Similar observations have been reported in the literature [44] associating thermal transitions between 220–230 °C with structural reorganization or the loss of crystalline water. Finally, the melting point of LEV is recorded at 235.7 °C.

In Figure 6b the thermograms of the LEV-loaded PLA and PLA-PE electrospun nanofibers are presented. While levofloxacin’s melting point is not directly observed, shifts in the glass transition (T_g_) and melting temperatures (T_m_) of both PLA and PLA-PE (presented earlier in Table 1) indicate drug–polymer interactions and demonstrate the effect of the drug on polymer properties. In all four cases, the observed lowering or broadening of the T_m_ peaks compared to pure polymers, the shifting of the glass transition temperatures to higher values, as well as the broad exothermic peaks associated with the cold crystallization, suggest that the incorporation of levofloxacin is not merely amorphously dispersed, as discussed earlier, but affects the macromolecular chain mobility and the polymer crystallization process. Moreover, the observed broadening of thermal peaks, particularly in the PLA-PE samples, may further suggest non-uniform drug distribution within the polymer matrix.

### 3.6. Estimation of Degradation Rate

**Figure 7:**
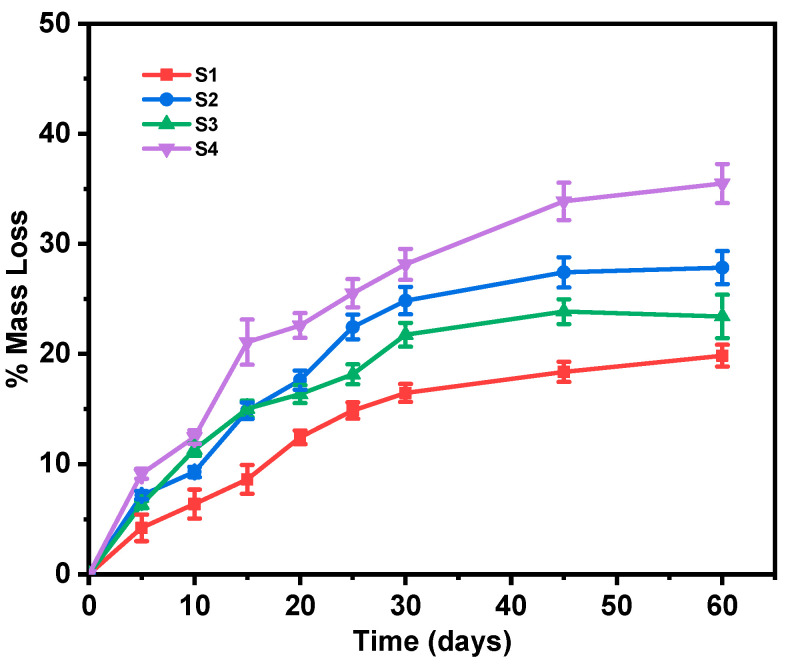
% Mass loss of the prepared nanofiber samples over a period of 60 days.

The degradation rate is another critical parameter that directly affects drug release, regardless of the release mechanism, i.e., matrix erosion or diffusion, and is therefore important to examine [47]. Figure 7 illustrates the percentage mass loss of the fabricated electrospun nanofiber samples over a 60-day degradation period. All samples exhibited a progressive mass loss, with a faster rate during the first 15–20 days of study (probably controlled by a surface erosion mechanism) which already led to almost half of their total mass loss, followed by a more sustained pattern that could be attributed to a bulk erosion process. While both PLA and PLA-PE nanofibers showed a similar mass loss pattern, the PLA-PE matrices (S2 and S4) demonstrated a higher degradation rate compared to the PLA matrices (S1 and S3), indicating that the introduction of pentaerythritol enhances the degradation susceptibility of the polymer. Moreover, the samples with 20% levofloxacin loading (S3 and S4) showed a substantially higher mass loss compared to the 10% levofloxacin samples (S1 and S2) for both used polymers, suggesting that higher drug concentrations accelerate the degradation process. This increased degradation could be attributed to drug-induced changes in the polymer matrix or increased porosity, facilitating faster water penetration.

### 3.7. In Vitro Drug Release Studies

The release of LEV from the electrospun fibers was performed in an environment simulating human body fluids (PBS buffer, pH 7.4). The drug release rate was evaluated and quantified using the analytical method described previously. Table 4 summarizes the drug loading and encapsulation efficiency values for the four studied LEV-loaded formulations. The % DL ranged from 17.91% to 34.78%, while % EE varied from 58.29% to 81.32%. Higher DL values were observed in formulations S2 and S4, both produced by PLA-PE, suggesting that the polymer composition influences the drug incorporation and retention within the nanofibers. The increased porosity of PLA-PE nanofibers, as discussed earlier, most likely contributes to these increased DL values, by providing more internal spaces for LEV incorporation. However, the lower EE values compared to PLA possibly indicate that some drug loss occurs during processing due to phase separation and/or drug migration to the fiber surface.

**Figure 8:**
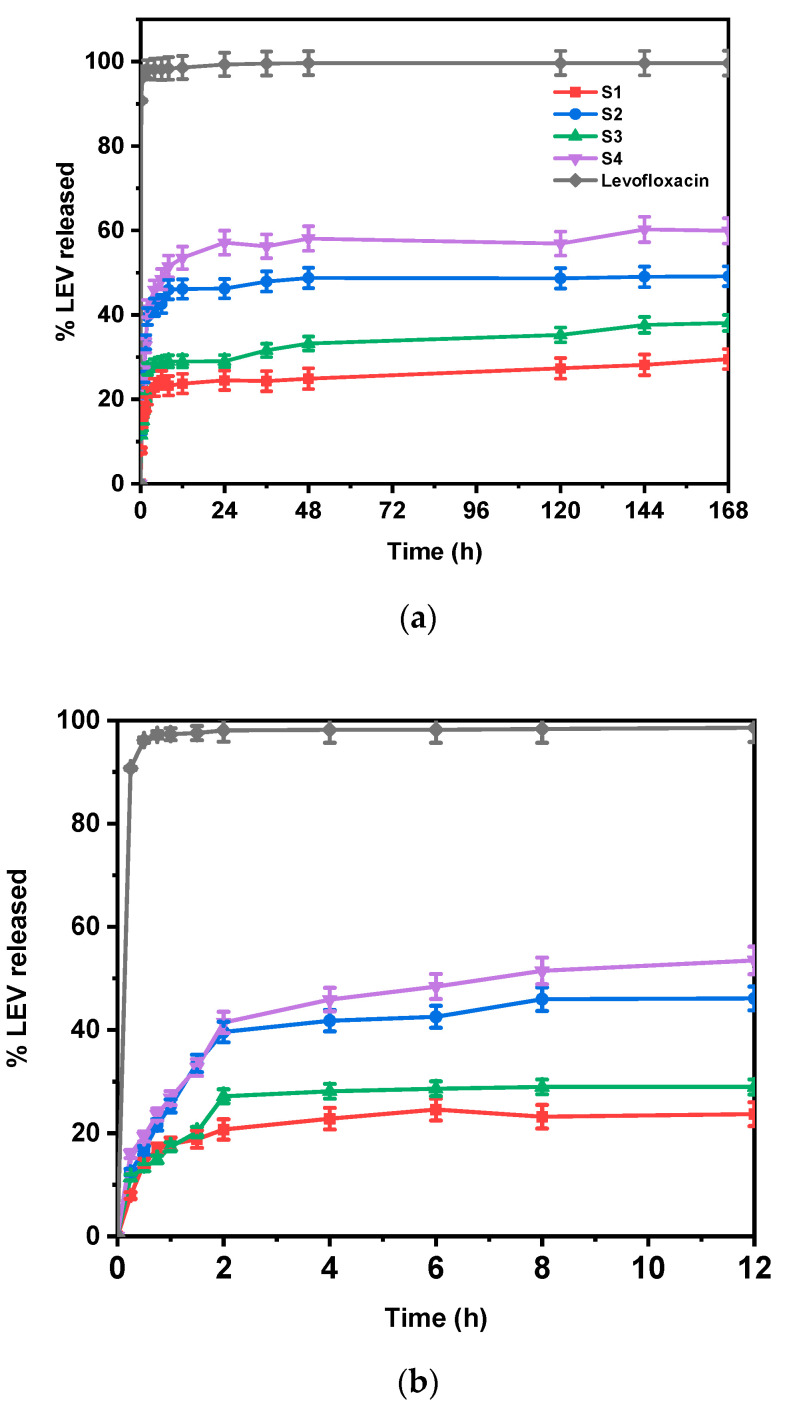
In vitro drug release profiles of all prepared nanofibers and pure levofloxacin (**a**) over a week and (**b**) during the first 12 h of study.

Figure 8a illustrates the drug release profiles of all prepared nanofibers and pure levofloxacin over a week, clearly showing that the polymer composition significantly impacts the release. Figure 8b provides a closer look at the drug release during the first 12 h, highlighting a significant burst release observed in all cases. This initial rapid release of LEV is likely due to the drug molecules located near the surface of the nanofibers and are very quickly released into the dissolution medium, as previously reported in the literature [48]. These data also match our SEM findings, where bead formation, especially in PLA-PE samples, implied possible drug-rich domains, which can potentially lead to a burst release effect [49]. The subsequent sustained release is attributed to the drug molecules embedded within the polymer nanofibers, which are released at a slower rate controlled by drug diffusion through the polymer matrix and polymer degradation. Comparing the LEV release profiles from the different nanofiber mats, it is evident that the drug release rate varies depending both on the polymer composition and the amount of drug loaded. Formulations S3 and S4, prepared with a 20% *w*/*w* of LEV, showed a faster release rate compared to S1 and S2 (10% *w*/*w* of LEV), an observation that aligns with the results on porosity calculations, where S3 and S4 exhibited higher porosity, which can facilitate drug diffusion, and therefore its release, by allowing more water penetration into the structure. In the same sense, the influence of the polymer matrix on drug release is also apparent. The PLA-PE nanofibers (S2 and S4) exhibited faster release rates and a higher percentage of LEV released (49.12 and 60.22%, respectively) compared to the PLA nanofibers (S1 and S3, achieving 29.5 and 38.1%, respectively), which could be attributed to their higher porosity and faster degradation rates, as previously discussed.

## 4. Conclusions

The present study successfully demonstrated the fabrication and comparative characterization of PLA and PLA-PE electrospun nanofibers for transdermal drug delivery. The PLA-PE copolymer led to nanofibers with enhanced properties, including higher porosity (90.1% vs. 82.4% for PLA) and larger fiber diameter (up to 665 nm). Degradation studies showed a faster mass loss in PLA-PE samples (~55%) compared to PLA (~43%) over 60 days. The incorporation of levofloxacin into the nanofibers was confirmed by FTIR spectroscopy, and the drug’s physical state within the nanofibers was shown to be amorphous by XRD analysis, which is favorable for drug dissolution and release. In vitro drug release profiles demonstrated biphasic release patterns of LEV from the nanofibers, with an initial burst release followed by a gradual release phase, whereas the solution composition (i.e., polymer and drug concentration) played a key role in the release profiles, showing that PLA-PE mats released significantly more LEV (up to 60.2%) than PLA (38.1%), supporting their utility for faster and more efficient drug delivery. To further refine the understanding of the release kinetics, future studies could employ mathematical modeling to analyze the release profiles and obtain specific kinetic parameters, although this was beyond the scope of our current investigation. To the best of our knowledge, this study marks the first use of PLA-PE copolymer in electrospinning, demonstrating its potential as an alternative to PLA for applications where controlled and accelerated drug release is desired.

## Figures and Tables

**Table 1:** Values of interest for the two polymers used in the present study.

Sample Name	Tm °C	Tg °C	Tc °C	[η] (dL/g)	Mn (g/mol)	PDI
PLA	175.9	56	116	1.32	55,500	2.18
PLA-PE	150	51	118	0.47	25,000	1.81

**Table 2:** Different conditions towards an optimized fabrication process.

Polymer	Polymer Concentration (% *w*/*v*)	Voltage (kV)	Rotation Speed (rpm)	Distance (cm)	Flow Rate (μL/h)
PLA	5	22	500	10	1000
	5	22	5000	10	1000
	**10**	**20**	**1000**	**12**	**750**
	10	20	1000	12	1000
	15	20	1000	12	750
	15	20	1000	12	750
PLA-PE	10	20	500	10	1500
	10	22	500	10	1500
	15	20	1000	12	1000
	15	22	1000	12	1000
	**20**	**20**	**1000**	**12**	**750**
	20	22	1000	12	750

**Table 3:** Solution concentrations and ES process parameters for the fabrication of the final electrospun PLA-based mats, and their calculated average fiber diameters.

Sample Name	Polymer Concentration (% *w*/*v*)	LEV Concentration (% *w*/*w*)	Voltage (kV)	Rotation Speed (rpm)	Flow Rate (μL/h)	Average Fiber Diameter (nm)
PLA10LEV10 (S1)	10	10	20	1000	750	334.8 ± 12.2
PLAPE20LEV10 (S2)	20	10	20	1000	750	526.3 ± 18.8
PLA10LEV20 (S3)	10	20	20	1000	750	442.7 ± 10.6
PLAPE20LEV20 (S4)	20	20	20	1000	750	665.2 ± 24.1

**Table 4:** % DL and EE values for the four studied LEV-loaded formulations.

Sample Name	% Drug Loading	% Encapsulation Efficiency
S1	17.91 ± 1.47	78.21 ± 0.82
S2	28.52 ± 1.93	64.28 ± 2.81
S3	22.32 ± 0.98	81.32 ± 1.27
S4	34.78 ± 2.81	58.29 ± 2.94

## Data Availability

The original contributions presented in this study are included in the article. Further inquiries can be directed to the corresponding author.
